# The proportion of genetic similarity for liability for neuroticism in mother–child and mother–father dyads is associated with reported relationship quality

**DOI:** 10.1038/s41598-025-14137-2

**Published:** 2025-08-12

**Authors:** Rebecca M. Pearson, Elizabeth C. Braithwaite, Tim Cadman, Iryna Culpin, Ilaria Costantini, Miguel Cordero, Marc H. Bornstein, Deborah James, Alex S. F. Kwong, Hannah Jones, Hannah Sallis

**Affiliations:** 1 School of Psychology, Faculty of Health and Education, Manchester Metropolitan University, Manchester, UK; 2 Section of Epidemiology, Department of Public Health, University of Copenhagen, Øster Farimagsgade 5, Copenhagen K, DK-1353 Denmark; 3 Centre for Academic Mental Health, Population Health Sciences, University of Bristol, Bristol, UK; 4 Department of Child and Family Health, Florence Nightingale Faculty of Nursing, Midwifery and Palliative Care, King’s College London, London, UK; 5 Instituto de Ciencias e Innovación en Medicina, Universidad del Desarrollo Facultad de Medicina Clínica Alemana, Las Condes, Chile; 6 Eunice Kennedy Shriver National Institute of Child Health and Human Development, Bethesda, MD USA; 7 Institute for Fiscal Studies, London, UK; 8 School of Education, Manchester Metropolitan University, Manchester, UK; 9 Division of Psychiatry, University of Edinburgh, Edinburgh, UK

**Keywords:** Neuroticism, Polygenic scores, Genetic similarity in neuroticism liability, Relationship quality, Marital conflict, ALSPAC, Behavioural genetics, Human behaviour

## Abstract

This study aims to explore the influence of genetic similarity for neuroticism liability in mother’s reported quality of relationship with her child and partner. Such understanding could provide insight into the role of genetic similarity in neuroticism liability in close relationships. Molecular genetic data in 4,704 mothers, partners, and children in the Avon Longitudinal Study Parents And Children (ALSPAC) study were used to derive the proportion of genetic similarity in neuroticism liability between mother and child, and mother and partner, for genetic variants associated with neuroticism. Associations between genetic similarity in neuroticism liability scores and mothers’ reported enjoyment and conflict in the parenting relationship (child ages 0–3) and her reported partner relationship were examined. For a one standard deviation (SD) increase in similarity in mother and child genetic variants associated with neuroticism, there was a 0.15SD (95%CI = 0.003 to 0.500, *p* = 0.046) increase in maternal reported enjoyment in their relationship. This association was greater where mother and child were both in the top quartile for high neuroticism (standardised beta = 0.29, 95%CI = 0.02 to 0.56, *p* = 0.034). Similar patterns of results emerged for similarity for genetic variants associated with neuroticism between mothers and partners, and the quality of the mother-partner relationship. These results highlight how phenotypic variation (i.e. the link between PGS scores and mothers reported enjoyment) linked to genetic liability in one individual may be linked with the genetic liability of those around them (i.e. the genetic liability of the infant). In other words, parenting and intimate partner relationships as perceived by the mother were explained not by one or the other’s genetic score, but by the similarity between them These exploratory findings present an intriguing mechanism by which similarity between genetic liability might be linked to family relationships.

**Supplementary Information:**

The online version contains supplementary material available at 10.1038/s41598-025-14137-2.

## Introduction

The quality of maternally-reported parent-child and parent-parent relationships have been linked to multiple child and parent outcomes, including mental health risk, well-being and academic achievement^1^. Understanding and facilitating family relationships that are perceived as enjoyable and fulfilling is therefore important. The sensitivity of parents to children’s emotional and mental state (mind-mindedness) is a hypothesised key factor linked to sensitive parent-child relationships^2^. Feeling understood is what most people want from relationships, including parent-child relationships^3^. The dominant focus of developmental research has been parental characteristics that influence mutual understanding in parent-child and parent-parent relationships, however, even in young children, parent-child interactions are increasingly found to be bidirectional and transactional^4^. Relationship research has also considered the role of personality factors in relationship satisfaction, and neuroticism has been associated with cognitive, emotional and behavioural processes in relationships^5^. Neuroticism is a personality trait which reflects the frequency and intensity of negative emotions^6^and recent research has identified genetic variants associated with neuroticism^7,8^. Here, we consider how mother, partner *and* child genetic similarity for neuroticism influences maternally-reported mother-child and mother-partner relationships. We chose neuroticism as a focus because our hypothesis is that emotional sensitivity is a core ingredient of relationships. Such insight may provide a new dimension to our understanding of the role of combined parent and child emotionality in close relationships, which is important in family-centred support.

The role of similarity (homophily) in attractiveness, mate selection and friendships is well established^9,10^. Although most of this work considers structural or physical similarities, it is also noted that value similarity (holding similar values and morals) is a key component in driving long-lasting relationships^11^. We also know that if an infant looks more like their father, then fathers are more involved in parenting^12^. This suggests that physical levels of similarity, which are known to play a part in mate selection and friendships^9,10^may also be important in evoking positive parent-child relationships. Further, personality similarity between parents and adolescents is associated with fewer internalising and externalising behaviours in adolescents, both concurrently and over time^13^.

There is good evidence that similarity in looks, personality and values, play an important role in relationship quality, however there is little knowledge of how emotional similarity may influence relationships. To investigate the role of emotional similarity in relationships, we consider the role of similarity in neuroticism. Neuroticism is one of five key personality traits and is defined as the propensity to experience *negative* emotions more frequently and intensely in response to stressors^14^. Neuroticism has a genetic component^15^and there is good evidence of a relationship between neuroticism and the functioning and structure of several emotion processing networks in the brain, particularly in response to negative stimuli^16^. Individuals high in neuroticism may require more emotional support, which may be more easily noticed and understood by those with similar levels of neuroticism. Indeed, higher neuroticism is linked to higher empathy and greater responses to distress in others^17^. Parental empathy has a greater impact on the pro-social behaviour of offspring with higher neuroticism^17^. Genetic liability for neuroticism is manifested in emotionality which can be detected in infants as early as 6 months^18^therefore the bidirectional nature of parent-child interactions means that neuroticism is likely to shape early parent-child relationships.

However, similarity in neuroticism may be shaped by the environment and genetics. Parents and infants who are highly attuned to each other may become more similar over time (i.e., the direction of the association is from parenting to similarity, and not vice-versa). One approach to separate out the causal role of similarity from potential social learning/contagion (when someone’s emotional and related behaviours lead to similar emotions and behaviours in others) of traits over time is to consider genetic similarity (a measure of the genetic relatedness among individuals for a specific phenotype^19^ in dyads or family groups. Genetic similarity can be measured using a variety of methods, including comparing DNA sequences, proteins, or other genetic markers^8,20^but to our knowledge cumulative similarity scores based on the proportion of similarity across a number of risk alleles for a specific personality trait has not been done before. Assessments of genetic similarity is a useful tool across a variety of academic research fields, such as evolutionary biology, medicine, forensic science, and anthropology. The key advantage of assessing genetic similarity rather than observed similarity in parenting research is that genetic similarity on a trait is determined at conception, and therefore genetic variants cannot be influenced by either the parent or the child over time, even if the manifestation of the trait can be. Parents and children are always approximately 50% genetically related, however, there will still be variation in how genetically similar they are on specific traits.

To our knowledge, no previous studies have examined parent-child *genetic* similarities; however, there is evidence from adoption and evocative genetic literature to draw on. The adoption literature provides an extreme example of parent-child genetic differences^21^and one study reports more conflict in adoptive dyads (compared to non-adoptive parents and children), which could not be attributed to adolescent age or ethnic minority status^22^. The polygenic prediction of educational attainment is different in adopted and non-adopted individuals^23^. There are of course alternative (especially socially/environmentally evoked) explanations for differences in adoptive parent-child relationships, but these studies are consistent with more genetic differences being linked to more challenges in relationships.

The role of children’s genetics on parental behaviour is also well established from evidence for child evocative genetic effects. Child evocative effects refer to the influence of genetic liabilities in the child on parental behaviour^24^. Previous work using family designs has provided evidence for evocative genetic effects: where genetic liability for behavioural problems (as inferred from biological family history), for example, is associated with harsher parenting in adoptive parents^25^. Experimental designs have also demonstrated that higher levels of conduct disorder in boys elicits more harsh parenting from mothers^26^. This study demonstrates that mothers’ harsh parenting responses are linked to child characteristics rather than mother’s shared family history and experiences. In addition, a recent twin study estimated that considerable variances in parental warmth (27%) and stress (45%) was attributable to child genetic influences on parenting^27^.

Parenting behaviour that is evoked by genetic liabilities is important in realising child potential (for good or bad)^28,29^. Thus, if evocative effects can be understood in the context of the parent’s liabilities, they may be more appropriately targeted in interventions to enhance good outcomes and prevent poor outcomes. For example, depending on the characteristics of the parent, and their emotional interpretations, a more defiant child may evoke either conflict or boundary setting, and a more neurotic child may evoke empathy and emotional scaffolding or avoidance and dismissal. Parental reaction may also be important for the eventual manifestation of the child defiance or neurotic liability and maybe important in more personalised interventions. Despite the potential roles of parent’s and children’s characteristics in influencing the direction and extent of evocative effects, the role of *similarity* in parent and child liability for emotional sensitivity has not yet been investigated.

We hypothesised that mothers with greater similarity in genetic liability for neuroticism to their child would be associated with the mothers higher reported enjoyment of relationships. To test this hypothesis, we utilised data from recent large genome wide association studies which have identified several genetic variants robustly associated with neuroticism, enabling the creation of a polygenic score (PGS) with good predictive power^7^. In addition to deriving a simple PGS, we can examine how similar parents and children are at each of the genetic variants and estimate a ‘proportion of similarity’, i.e., how may risk alleles parents and children share. We can also explore direct associations between mother and child neuroticism genetic liability scores and parenting, to disentangle whether it is the trait itself or *similarity* in the trait that is related to variation in the parent’s reported enjoyment of the relationship. There are for example, links between maternal neuroticism and depressed mood and low parental-reported enjoyment in parent-child relationships^1^.Thus,, if low neuroticism liability is associated with more enjoyment, any association with similarity may partially reflect that both mother and child have lower genetic liability for neuroticism. This is important because there is also evidence that neuroticism and parenting are related^30^. Overall levels of neuroticism in both parent and child may also influence any impact of similarity. For example, it could be argued that two individuals with high neuroticism are quite different from two individuals with low neuroticism. However, if emotional understanding and empathy is the causal ingredient, then being *similar* at either end of the spectrum is what is important. We also examine associations with similarity according to child sex. We hypothesised that similarity scores may be more important for mothers and sons because emotional expression can be sex specific^31^ and therefore harder to understand across different sex pairs. Additionally, we examine whether maternally-reported mother-partner similarity is also linked to mothers’ relationship with her child. For example, similarity in the infant may be a proxy for similarity to the partner, and mothers may for example feel more warmth towards a child that reminds them of their chosen life partner, but this may be dependent on the intimate partner relationship quality.

To further understand the specificity of our findings, we proposed two further scenarios: one in which we expect to replicate the associations (positive control) and one in which we do not (negative control). First, we explored whether associations between the proportion of similarity for genetic liability for neuroticism and relationship quality extends to adult relationships by examining similarity in genetic liability for neuroticism in mothers and partners and the maternally-reported marital relationship. Second, we include a negative control; by examining the genetic similarity in rheumatoid arthritis, a trait which does not share the proposed causal mechanism of facilitating emotional understanding but was derived using the same methods. Our hypotheses are as follows:

1. Mothers who have a greater proportion of similarity in genetic liability for neuroticism with their child will report more enjoyment and less conflict in the relationship.
2. Mothers who have a greater proportion of similarity in genetic liability for neuroticism with their partners will report higher enjoyment in parenting, but this will be explained by the inter-partner relationship.
3. Mothers who have a greater proportion of similarity in genetic liability for neuroticism with their partner will report a higher quality of the inter-partner relationship. 4. There will be no association between the mother’s genetic similarity in rheumatoid arthritis with her child and maternally-reported enjoyment of mother-child and mother-partner relationships.
4. There will be no association between the mother’s genetic similarity in rheumatoid arthritis with her child and maternally-reported enjoyment of mother-child and mother-partner relationships.

## Methods

### Participants

The sample comprised participants from the Avon Longitudinal Study of Parents and Children (ALSPAC), an ongoing population-based study. Pregnant women resident in Avon, UK with expected dates of delivery between 1st April 1991 and 31st December 1992 were invited to take part in the study. 20,248 pregnancies have been identified as being eligible and the initial number of pregnancies enrolled was 14,541. Of the initial pregnancies, there was a total of 14,676 foetuses, resulting in 14,062 live births and 13,988 children who were alive at 1 year of age. When the oldest children were approximately 7 years of age, an attempt was made to bolster the initial sample with eligible cases who had failed to join the study originally. As a result, when considering variables collected from the age of seven onwards (and potentially abstracted from obstetric notes) there are data available for more than the 14,541 pregnancies mentioned above: The number of new pregnancies not in the initial sample (known as Phase I enrolment) that are currently represented in the released data and reflecting enrolment status at the age of 24 is 906, resulting in an additional 913 children being enrolled (456, 262 and 195 recruited during Phases II, III and IV respectively). The phases of enrolment are described in more detail in the cohort profile paper and its update^32–34^. The total sample size for analyses using any data collected after the age of seven is therefore 15,447 pregnancies, resulting in 15,658 foetuses. Of these 14,901 children were alive at 1 year of age. Of the original 14,541 initial pregnancies, 338 were from a woman who had already enrolled with a previous pregnancy, meaning 14,203 unique mothers were initially enrolled in the study. As a result of the additional phases of recruitment, a further 630 women who did not enrol originally have provided data since their child was 7 years of age. This provides a total of 14,833 unique women (G0 mothers) enrolled in ALSPAC as of September 2021. G0 partners were invited to complete questionnaires by the mothers at the start of the study and they were not formally enrolled at that time. 12,113 G0 partners have been in contact with the study by providing data and/or formally enrolling when this started in 2010. 3,807 G0 partners are currently enrolled. The sample for the current study comprises 4,704 mothers and offspring who had complete genetic and parenting data available for the analyses described below.

### Measures

#### Parenting

We used extracted factor scores from derived parenting factors previously reported in Culpin et al. (2020)^1^which fully details the item section and development of the parenting factors. In brief, potential items were extracted from self-reported questionnaires administered to mothers from pregnancy to age 3 years capturing parenting behaviour, attitudes and knowledge. Items categorised as parental enjoyment, conflictual relationships, and stimulation and teaching (based on parenting taxonomies) were extracted and entered into separate single-factor Confirmatory Factor Analysis (CFA) models. We focused on ages 0–3 years to capture a period of time when most mothers spend time with their children prior to the commencement of nursery school^24^. The relevant domains for our hypotheses here were enjoyment and conflict. Scores were derived from saved factor scores for each latent factor. A model using CFA to fit the following 3 factors with the following items showed good model fit. The RMSEA (0.024, 95%CI = 0.024–0.025) and the CFI (0.92) indicated that the measurement model fit the data well. More details on the parental enjoyment and conflict factors are available in Supplement 1. To provide predictive validity of the enjoyment factor we looked at how this factor related to the ‘child’s’ retrospective reports of feeling loved as a child, which they reported on at age 24 (n = 1,965). For a one unit increase in the maternal-reported enjoyment score at age 0–3 years, the child is 2.63 times more likely to report that they felt loved at age 24 (95%CI = 1.49 to 4.65, p = 0.001), this remains after adjusting for maternal age, child gender, and other dimensions of parenting such as stimulation.

#### Inter-partner conflict in the mother-partner relationship

Mothers’ self-report of inter-parental conflict was assessed using the Conflict Tactics Scale Revised (CTS2)^35,36^. This is a 39-item scale in which partners in a dating, cohabiting or marital relationship engage in psychological and physical attacks on each other and also their use of reasoning or negotiation to deal with conflicts. The questions are designed to be asked about both the participant and the partner which results in two questions for each item, and a total of 78 questions. Higher scores on this scale indicate more reported conflict. The CTS2 has been reported to be a reliable and valid instrument used across different populations and cultures^37^. In this sample, the mean score on the scale was 21.61 (Standard deviation = 7.25, range = 10–49, *n* = 10,514).

#### Polygenic scores for neuroticism

Genotyped data were available on 8,237 children and 8,196 mothers in the ALSPAC study. Data were also available on a small subset of partners in the cohort (*n* = 1,722). Full details of genotyping and quality control measures are available in Supplement 1, population stratification methods were applied at this stage and analyses are based on individuals of European descent. From the original genome wide association study (GWAS), 116 independent variants were found to be robustly associated with neuroticism^7^. Of these original variants, 109 were available in ALSPAC. Weighted PGS for neuroticism were calculated for each mother, partner and child in ALSPAC with genetic data using PRSice2^38^. These are calculated as the sum of the number of copies of each effect allele carried by an individual (this ranged from 0 to 2 for each SNP), multiplied by the effect estimate identified in the original GWAS. These weighted sum scores were then standardised prior to use in the analysis. Correlations between PGS from individuals were: mother and child *r* = 0.49, father and child *r* = 0.47, mother and father *r* = 0.05.

#### Proportion of similarity in neuroticism PGS in relationship pairs

A proportion of similarity measure was calculated by counting the number of neuroticism SNPs at which mothers and offspring, and mothers and partners, had the same genotype, see Table 1. This proportion was then divided by the total number of neuroticism SNPs to give the proportion of neuroticism variants at which mothers and offspring/partner shared a genotype. This proportion of similarity was available for 5,470 mother-offspring pairs and ranged from 0.46 to 0.80. Of these, 4,704 mothers and offspring had complete genetic and parenting data and comprise the sample used for all analyses described below. The partner proportions of similarity were available on a subset of 1,292 couples and ranged from 0.34 to 0.70.

**Table 1:** Worked example of creation of the similarity score, with 8 SNPS.

	Number of risk alleles at each SNP		Parent and child genotypes identical?
	Parent	Child	
SNP_1_	0	1	No
SNP_2_	1	1	Yes
SNP_3_	1	2	No
SNP_4_	2	2	Yes
SNP_5_	1	0	No
SNP_6_	2	1	No
SNP_7_	2	1	No
SNP_8_	1	1	Yes
Proportion of identical genotypes	3/8 = 0.375		

### Data analysis

The sample of mothers with complete genetic data was compared on several demographic variables with those for whom genetic data were missing using chi-squared tests. Associations between PGS, proportion of genetic similarity for neuroticism scores, and maternal reports of enjoyment of parenting, conflict and marital relationship were conducted using linear regression models in Stata version 17. Linear regression was chosen given that all variables were continuous and normally distributed. PGS and similarity scores were continuous exposure variables, and extracted parenting factors and marital relationships were continuous outcome variables. Associations were also examined by child sex.

First, we examined main effects of mother and child PGS for neuroticism on maternal-reported enjoyment and conflict of the mother-child relationship. Next, we examined the association between proportion of similarity in mother-child PGS for neuroticism with maternal-reported enjoyment and conflict in the mother-child relationship. To understand whether the degree of neuroticism (i.e., both members of the dyad being high or both low) might influence maternal reports of enjoyment with their child, we investigated the association between the proportion of similarity between mother and child for neuroticism PGS and maternal enjoyment, stratified by groups according to mother and child both being in the top or bottom 50% for PGS for neuroticism. Next, we examined associations between mother-partner similarity in PGS for neuroticism and maternal-reported enjoyment and conflict in the mother-child relationship. In this model, we also included maternal-reported marital conflict as a confounder in an additional step to examine whether such association might be explained by conflict in the marital relationship.

In addition, we also performed two sensitivity analyses. For our positive control, we calculated a proportion of similarity for neuroticism between mothers and partners and examined the association with maternal-reported inter-partner conflict. Again, to understand whether the degree of neuroticism (i.e., both members of the dyad being high or both low) might influence maternal reports of the inter-partner conflict, we investigated the association between the proportion similarity across all neuroticism SNPs between mother and partner and marital conflict, stratified by groups according to mother and partner both being in the top or bottom 50% for PGS for neuroticism.

As a negative control, we calculated a weighted PGS for Rheumatoid Arthritis (RA) as described above. From the original GWAS, 101 independent variants robustly associated with RA were identified^39^and of these 87 were available in ALSPAC. We also used these genetic variants to calculate a measure of mother-child proportion of similarity (i.e. the proportion of these SNPs for which mothers and children shared the same genotype). This was available for 5,470 mother-child pairs and ranged from 0.45 to 1.00. We examined the association between the RA similarity measure and maternal reported enjoyment and conflict in the mother-child relationship.

## Results

Mothers for whom complete genetic data were available for analysis were compared with those who did not have complete data, and descriptive results are displayed in Table 2. Mothers with complete genetic data had higher educational qualifications (chi^2^ = 192.83, *p* < 0.001), were more likely to be married or co-habiting (chi^2^ = 112.72, *p* < 0.001), had fewer children at the time of pregnancy (when recruited to the study) (chi^2^ = 53.26, *p* < 0.001), and were less likely to have ever smoked (chi^2^ = 43.51, *p* < 0.001) than mothers for whom genetic data was missing.

**Table 2:** Descriptive data for mothers for whom complete genetic data was available and were included in analyses, and those with missing genetic data.

Variable	Cases with missing data		Complete case for analysis	
	*N*	%	*N*	%
Mothers highest educational qualification				
CSE	603	22.13	575	12.46
Vocational	287	10.53	371	8.04
O-level	960	35.23	1589	34.44
A-level	549	20.07	1258	27.26
University degree	313	11.49	809	17.53
Mothers marital status				
Never married	570	19.68	571	12.28
Widowed	5	0.17	6	0.13
Divorced	138	4.76	154	3.31
Separated	57	1.97	49	1.05
1st marriage	1930	66.62	3519	75.66
2nd or 3rd marriage	172	5.94	329	7.07
Mothers parity				
Zero	1168	40.51	2146	46.19
One	1074	37.23	1628	35.04
Two or more	589	20.42	825	17.76
Mother ever smoked				
Yes	1497	51.93	2073	44.62
No	1364	47.31	2553	54.95

### Main effect of mother and child PGS for neuroticism on maternal-reported enjoyment of parenting and conflict

There was weak evidence that increased maternal (Beta = 0.007, 95%CI=-0.000 to 0.014, *p* = 0.060) and child (Beta = 0.008, 95%CI = 0.000 to 0.015, *p* = 0.030) PGS for neuroticism was associated with higher maternal-reported conflict, and no evidence of a correlation with enjoyment for mothers (Beta = 0.005, 95%CI=-0.003 to 0.012, *p* = 0.213) or children (Beta = 0.004, 95%C I=-0.0035 to 0.011, *p* = 0.301). Partner PGS for neuroticism was not associated with either conflict or enjoyment in the maternal reported mother-child relationship.

### Proportion similarity: a higher proportion similarity between mother and child is associated with enjoyment of parenting, especially for boys

**Fig. 1:**
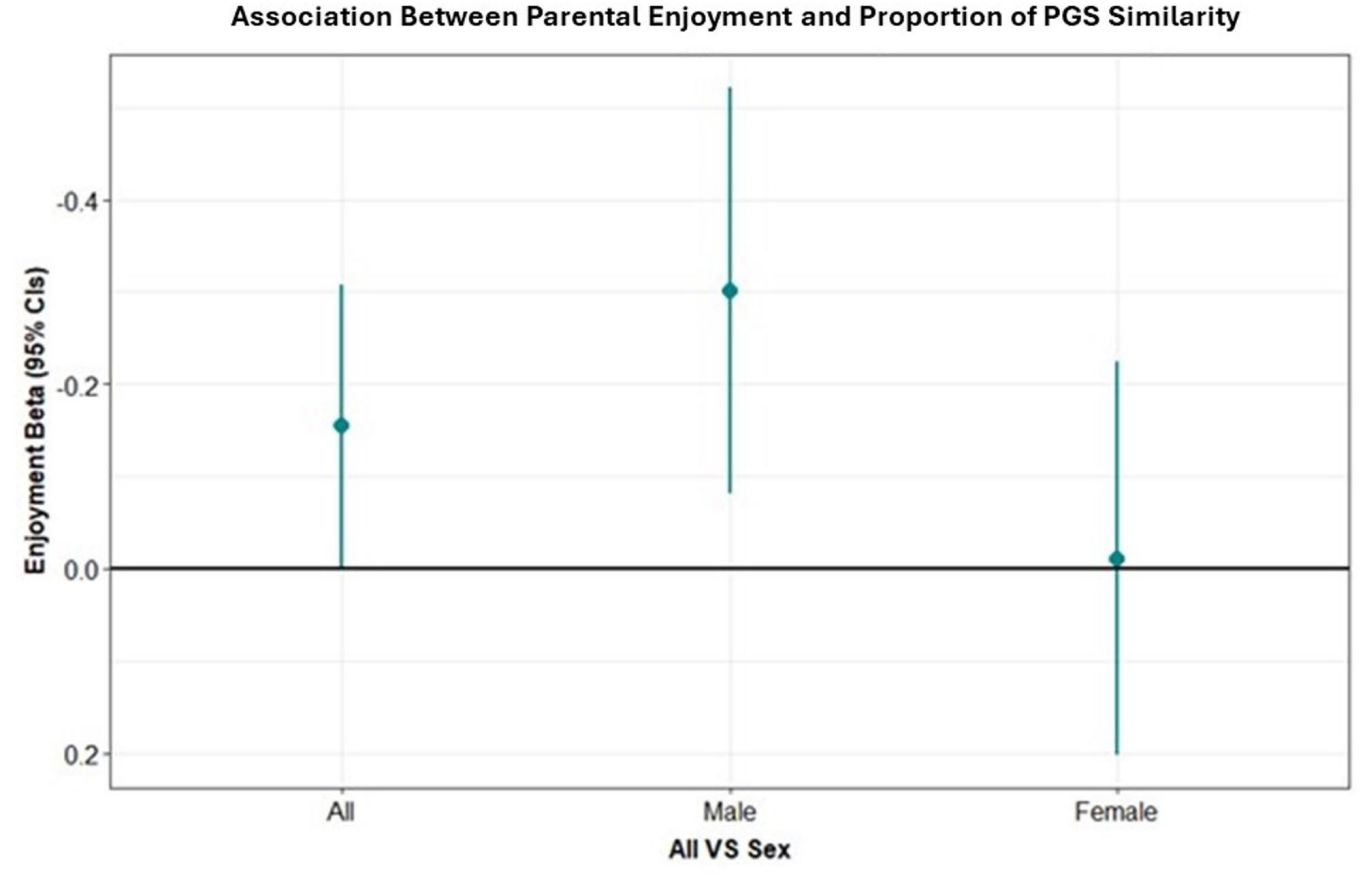
Beta coefficients representing the point increase in parental enjoyment for a 1 unit increase in the proportion of similarity in neuroticism PGS.

For a 1 standard deviation (SD) increase in genetic similarity in neuroticism liability between mother and child there was 0.15 SD (95% CI = 0.003 to 0.50, *p* = 0.046) increase in maternal-reported enjoyment of parenting (*N* = 4,704), displayed in Fig. 1. There was no evidence for an association with conflict (-0.09SD, 95%CI=-0.24 to 0.06, *p* = 0.251), although the association was in the expected direction, with increased similarity across the neuroticism SNPs being associated with less conflict. To further explore the association with enjoyment, we investigated the association split by child sex and found that the association was evident in mother-son dyads (Beta = 0.30 95%CI = 0.08 to 0.52, *p* = 0.007, *n* = 2,323) but not in mother-daughter dyads (Beta = 0.01, 95%CI= -0.20 to 0.22, *p* = 0.922, *n* = 2,374), see Fig. 1.

To understand whether the degree of neuroticism (i.e., both members of the dyad being high or both low) might influence maternal reports of enjoyment with their child, we investigated the association between the proportion similarity across neuroticism SNPs of mother and child and maternal enjoyment, stratified by whether mother and child were both in the top or bottom 25% for neuroticism PGS. As can be seen in Table 3 the results were strongest in dyads who were *both high* for neuroticism (*n* = 1,552).

**Table 3:** Associations between similarity in mother-child genetic liability for neuroticism and maternal reported enjoyment in the mother-child relationship where both mother and child are in the top quartile (high) for neuroticism PGS and both are in the bottom (low) quartile for PGS.

	Both mother and child top quartile for neuroticism PGS	Both mother and child bottom quartile neuroticism PGS
Association of PGS similarity and maternal-reported enjoyment	0.29 (0.02–0.56) *p* = 0.034	0.10 (0.16–0.37) *p* = 0.446
N	1552	1572

Standardised Beta, 95% Confidence Interval, and p-value displayed for each group.

We additionally conducted a sensitivity analysis to examine whether associations between mother-child similarity in genetic liability for neuroticism and maternal-reported enjoyment of the relationship were similar where mothers were experiencing very severe symptoms of depression (score of > 17 on the EPDS, *n* = 98). We find that there is a strong association in this group, with an effect size almost 10-fold higher than the association for the full population. In this group, there is a 1.4 SD increase in maternal reported enjoyment for each unit increase in proportion of genetic similarity, but with wide confidence intervals due to small participant numbers (95%CI = 0.0004 to 2.82, *p* = 0.05).

### Proportion similarity in neuroticism variants between mothers and fathers is associated with maternal-reported enjoyment of parenting, again, especially for boys

There was weak evidence to suggest that similarity scores between the mother and partner was associated with maternal-reported enjoyment of the mother-child relationship (Beta = 0.286, 95%CI = 0.005 to 0.578, *p* = 0.054). When split by sex, the association was stronger for maternal-reported enjoyment of their relationship with their child in mother-son dyads (Beta = 0.421, 95%CI = 0.000 to 0.841, *p* = 0.049), compared with mother-daughter dyads (Beta = 0.123, 95%CI=-0.275 to 0.523, *p* = 0.544). However, when inter-partner conflict was included in the regression models all associations of similarity across mother-partner neuroticism SNPs with enjoyment of mother-child relationship substantially attenuated for mother-son relationships (Beta=-0.300, 95%CI=-0.704 to 0.103, *p* = 0.145), but not mother-daughter relationships (Beta=-0.145, 95%CI=-0.523 to 0.234, *p* = 0.543). Proportion similarity between mother-partner neuroticism SNPs was not associated with mother-child conflict.

### Sensitivity analysis

#### Positive control: proportion of similarity in mother-partner neuroticism variants and quality of marital relationship

There was weak evidence that high similarity scores between mother and partner were associated with higher quality marital relationships, as reported by the mother (Beta = 0.986, 95%CI = 0.046–2.014 *p* = 0.061). Consistent with the mother-infant findings, there was a similar pattern in the same direction for mother reported quality of relationship for the individuals who were similarly high in genetic liability to neuroticism to their partner reporting higher relationship quality (Beta = 1.855, 95%CI=-0.319 to 4.029, *p* = 0.09) compared to both being low (Beta = 1.246, 95%CI=-0.899 to 3.391, *p* = 0.252). In the mother-partner pairs with high similarity scores, the estimate is stronger, and the p-value is lower, however the evidence of higher maternal reported relationship quality in these pairs compared to those with a low genetic liability scores is inconclusive as the CI’s overlap.

#### Negative control: proportion of similarity in mother-child variants for rheumatoid arthritis and maternal-reported enjoyment/conflict in the mother-child relationship

**Fig. 2:**
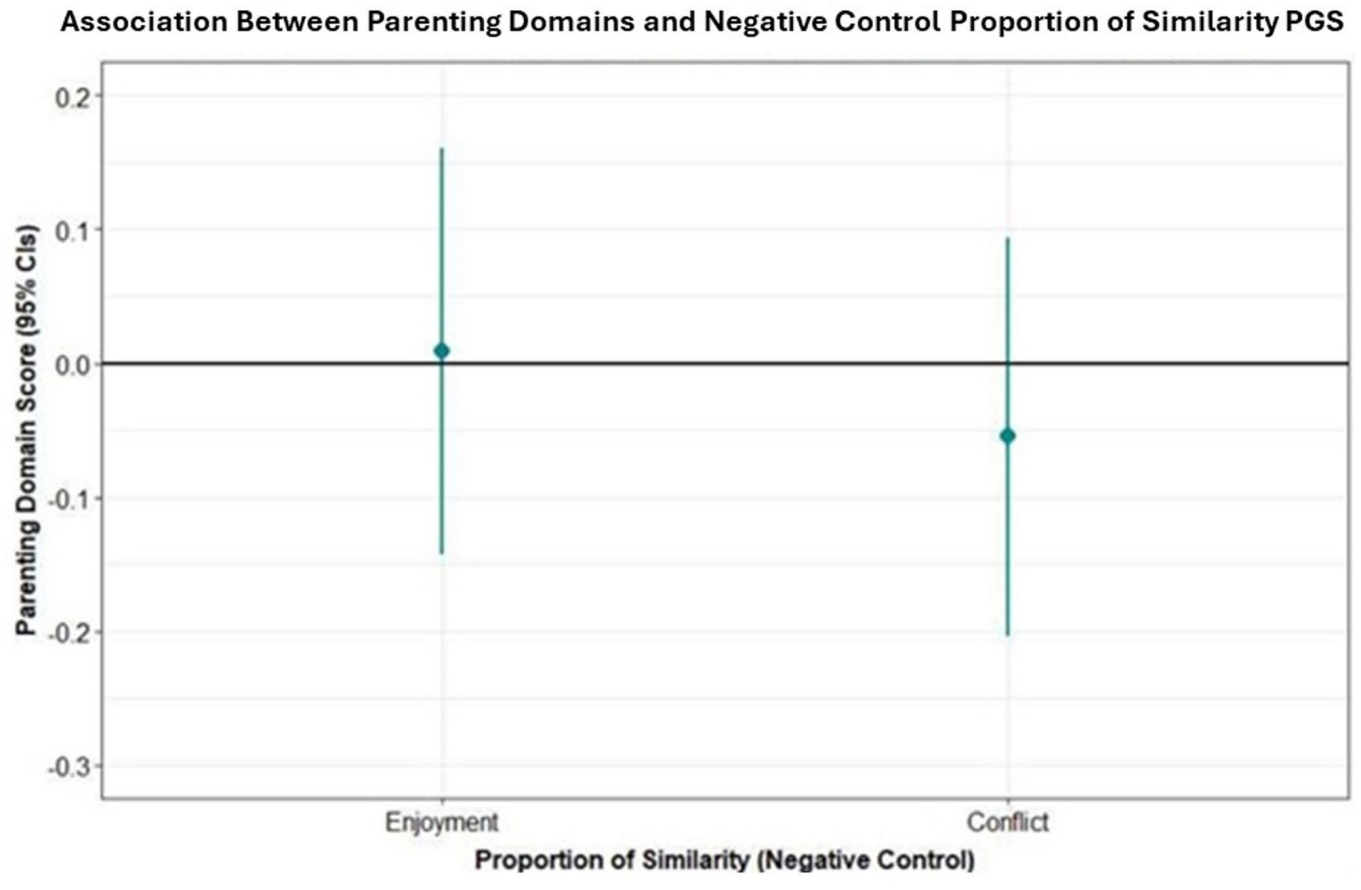
Beta coefficients representing the point increase in parental enjoyment for a 1 unit increase in the proportion of similarity in RA PGS.

There was no evidence for an association between maternal-child similarity for RA and maternal-reported enjoyment of parenting (Beta = 0.01, 95%CI=-0.14 to 0.16, *p* = 0.910) or conflict (Beta=-0.05, 95%CI=-0.2 to 0.09, *p* = 0.465), see Fig. 2.

## Discussion

We report that the *proportion of similarity* in genetic liability for neuroticism between mother and child was associated with higher maternal-reported enjoyment in the mother-child relationship, specifically in mother-son dyads. We also found that the association between genetic similarity in neuroticism liability and maternal-reported enjoyment was stronger in dyads where both the mother and child were in the top 25% of genetic liability for neuroticism. This suggests that it is unlikely to be the case that the association between genetic similarity in neuroticism liability and enjoyment result from low emotionality and associated calmness. Our findings are therefore more consistent with the importance of emotional understanding in relationship quality. We also found that the association may be stronger for mother-son relationships. One possibility is that emotional similarity and empathy are even more important when there is already a sex difference in a dyad, given known differences in emotional expression in boys and girls^31,40^. We report a similar pattern of results in partner relationships, suggesting that the same mechanism (for example, emotional understanding or similarity) may be important in adult relationships. Parenting (both conflict and enjoyment) and inter-partner conflict were reported by the mother. Therefore, the associations with child or partner genetic scores on maternal-reported parenting cannot be a reflection of trait and reporting of parenting (as the child did not report it).

We investigated similarity in genetic liability, which may not actually represent similarity in the manifested trait. What genetic liability for neuroticism means, and how it manifests in a young infant, is especially unclear^41^. Nonetheless, lack of understanding as to how it manifests, does not negate that whatever genetic liability to neuroticism does represent was associated with enjoyment in parent-child relationships and reduced conflict in the intimate partner relationship, as reported by the mother. In addition, here we investigated genetic liability for neuroticism at a population level and along a continuum. Neuroticism alone is not synonymous of mental health problems and indeed can represent emotional strength. However, more extreme levels of neuroticism that may be key in psychopathology may manifest differently. For example, high neuroticism in severe mental health presentations can be linked with an inability to regulate emotion which may cause conflicts^42^. However, we did conduct a sensitivity analysis with a sub group of mothers with very severe symptoms of depression^43^and found that associations between maternal-reported enjoyment in the relationship and genetic similarity for neuroticism were almost 10-fold greater in this group than in the full population. Thus, the associations between genetic similarity for neuroticism and maternal perceived enjoyment of relationships may be *qualitatively* different in the context of more severe mental health problems (to which high neuroticism is linked) where emotional understanding is especially hard. This could potentially be explained by Meltzoff’s concept of ‘like me’^44,45^which emphasizes how a sense of similarity is key to early connections between parents and infants. The capacity to draw on similarities may be especially critical when a mother’s capacity to experience enjoyment is depleted. During an episode of depression, if a mother can easily see her baby is like her in some way (either physically or emotionally) she may find it less effortful to connect with the baby. It may be that connectedness is the light that punctures the darkness of depression, and the emotional similarity may well be apparent in early parent-infant interactions.

Limitations of the study include a relatively small sample size and low variance in the trait explained by genetic liability, possibly resulting in low statistical power. We therefore report and evaluate exact p-values and strength of the evidence rather than arbitrary thresholds throughout. Triangulation of findings of similar patterns in mother-father dyads, and no associations with similarity scores for the negative control scenario, provide some confidence that this is not a chance finding. Nonetheless, the results require replication in a different, larger cohort due to low sample size and multiple comparisons. This is particularly the case for some of the stratified analyses which had very large confidence intervals. Additionally, we did not have information on partner-reported enjoyment in partner-child relationships and so were unable to conduct a parallel analysis in partner-child dyads, however this is an interesting area for future work.

### Implications

Genetics are not deterministic, they are what could be, not what is. Here we have identified a possibility of how mutually evocative genetic associations may link to the quality of relationships as perceived by the mother. We provide some initial evidence that genetic liability of a child is associated with parental relationships, and that genetic *similarity* in neuroticism liability between two individuals influences their relationship. These findings highlight why we should not look at individuals’ genetic liabilities in isolation. Rather, we should consider how one individual’s genetic liability for a trait is manifested may be influenced by genetic liabilities of those around them.

The associations we report are small, and the meaning of the effect sizes are difficult to interpret, but in terms of standardised differences the effects are around 0.1, which is considered small according to traditional indices^46^. Additionally, genetic scores only account for approximately 5% of variance in the neuroticism trait. Thus, other parental and child factors, both genetic and environmental, must also explain variance in parenting and child outcomes. It will be important for future work to consider how parent-child genetic similarity related to other personality traits, such as extraversion, is related to quality of parent-child relationship. Nonetheless, our findings suggest that similarity in genetic predisposition for emotional reactivity is likely important for parenting. If replicated, this implies that interventions to help understand emotional needs of children, as well as understanding that these may not be the same as one’s own, could be important in establishing greater enjoyment and warmth in parent-child relationships and emphasises the importance of more family-centred approaches. Personalised parenting interventions, which include micro-analysis of parent-child relationships are now more commonplace. Our findings, with replication, could be used to heighten the attention of the analyst (parent practitioner) to the mutual and bi-directional affordances that expressions of negative emotions are exerting for the benefit of emotional understanding, rather than focusing on the ‘negative’ emotion per se.

## Supplementary Information

Below is the link to the electronic supplementary material.

Supplementary Material 1

## Data Availability

ALSPAC data access is through a system of managed open access, for more information please see http://www.bristol.ac.uk/media-library/sites/alspac/documents/researchers/data-access/ALSPAC_Access_Policy.pdf.
