# Supplementary material for: The proportion of genetic similarity for liability for neuroticism in mother–child and mother–father dyads is associated with reported relationship quality
Source: Sci Rep. 2025 Aug 12;15:29505. doi: 10.1038/s41598-025-14137-2 (PMC12343813; doi:10.1038/s41598-025-14137-2)
Supplement: Supplementary file 1 — Supplementary Material 1 [file 41598_2025_14137_MOESM1_ESM.docx]

***Supplemenary information***

**Details on the parental enjoyment and conflict factors used in analyses.**

*Factor 1: Parental Enjoyment.* Parental enjoyment contains 14 items relating to enjoyment of the child from ages 4 weeks to 3 years 11 months (e.g., ‘I really enjoy my baby’, ‘Having a baby has made me feel more fulfilled’) as well as items relating to frequency of cuddling and playing with the child. Initially, items relating to feelings of irritation with the child (e.g., ‘This child gets on my nerves’) were included; however, in the final model they loaded better on the factor encapsulating conflictual mother-child relationship. The internal consistency of parental enjoyment is α=0.82 which indicates optimal internal consistency, with the summed items forming a normally distributed scale. Higher scores indicate more enjoyment. In the sample with genetic similarity data the mean enjoyment factor score was -0.004, SD 0.26, the distribution is shown in Supplementary Figure 1.


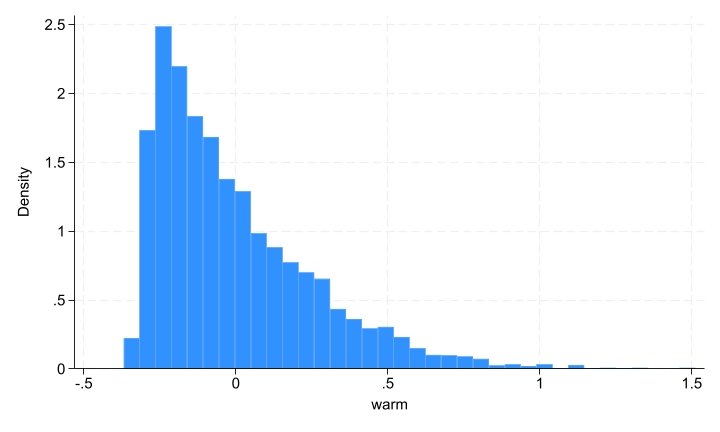


Supplementary Figure 1 Distribution of scores on the parental enjoyment score.

*Factor 2:* *Conflictual Relationships.* The conflictual parenting factor established in prior work^1^ contains 16 items relating to conflict, harsh discipline and irritation with the child (e.g., frequency of arguments, ‘battle of wills’, smacking and shouting) from ages 1 year 6 months to 3 years 11 months. At age 1 year 6 months, a substantial proportion of mothers reported having battles of wills (37%) and frequent conflict (21%) with their children. In addition, 24% of mothers reported having smacked their children sometimes during tantrums, whilst 58% of mothers reported having shouted at their child. At age 3 years 11 months, 17% of mothers reported that the child gets on their nerves. The internal consistency of conflictual relationships is α=0.75. Higher factor scores signify more conflictual relationships. In the sample with genetic similarity data the mean conflict factor score was 20.002, SD 0.25, the distribution is given in Supplementary Figure 2.

*
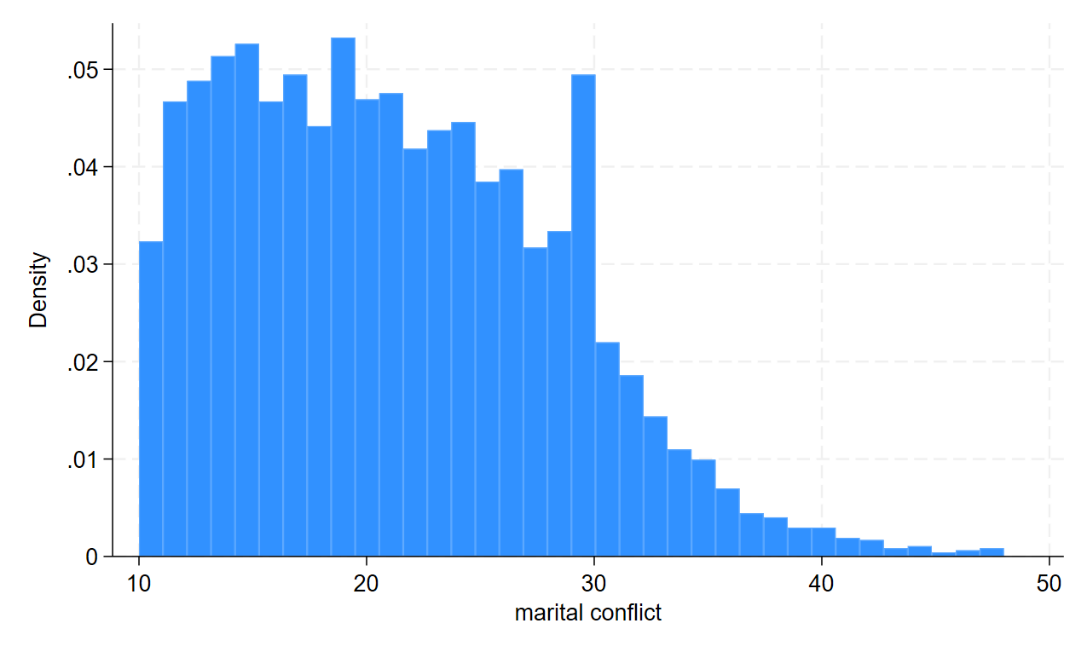
*

Supplementary Figure 2 Distribution of scores on the conflictual relationship score.

**ALSPAC genetic data generation and quality control**

ALSPAC children were genotyped using the Illumina HumanHap550 quad chip genotyping platforms. The resulting raw genome-wide data were subjected to standard quality control methods. Individuals were excluded on the basis of gender mismatches; minimal or excessive heterozygosity; disproportionate levels of individual missingness (>3%) and insufficient sample replication (IBD < 0.8). Population stratification was assessed by multidimensional scaling analysis and compared with Hapmap II (release 22) European descent (CEU), Han Chinese, Japanese and Yoruba reference populations; all individuals with non-European ancestry were removed. SNPs with a minor allele frequency of < 1%, a call rate of < 95% or evidence for violations of Hardy-Weinberg equilibrium (P < 5E-7) were removed. Cryptic relatedness was measured as proportion of identity by descent (IBD > 0.1). Related subjects that passed all other quality control thresholds were retained during subsequent phasing and imputation. 9,115 subjects and 500,527 SNPs passed these quality control filters.

ALSPAC mothers were genotyped using the Illumina human660W-quad array at Centre National de Génotypage (CNG) and genotypes were called with Illumina GenomeStudio. PLINK (v1.07) was used to carry out quality control measures on an initial set of 10,015 subjects and 557,124 directly genotyped SNPs. SNPs were removed if they displayed more than 5% missingness or a Hardy-Weinberg equilibrium P value of less than 1.0e-06. Additionally, SNPs with a minor allele frequency of less than 1% were removed. Samples were excluded if they displayed more than 5% missingness, had indeterminate X chromosome heterozygosity or extreme autosomal heterozygosity. Samples showing evidence of population stratification were identified by multidimensional scaling of genome-wide identity by state pairwise distances using the four HapMap populations as a reference, and then excluded. Cryptic relatedness was assessed using a IBD estimate of more than 0.125 which is expected to correspond to roughly 12.5% alleles shared IBD or a relatedness at the first cousin level. Related subjects that passed all other quality control thresholds were retained during subsequent phasing and imputation. 9,048 subjects and 526,688 SNPs passed these quality control filters.

After combining genotype data in the mothers and the children, SNPs with genotype missingness above 1% were removed due to poor quality (11,396 SNPs removed) and a further 321 subjects were removed due to potential ID mismatches. This resulted in a dataset of 17,842 subjects. Imputation of the target data was performed using Impute V2.2.2 against the 1000 genomes reference panel (Phase 1, Version 3) (all polymorphic SNPs excluding singletons), using all 2186 reference haplotypes (including non-Europeans).

This gave 8,237 eligible children and 8,196 eligible mothers with available genotype data after exclusion of related subjects using cryptic relatedness measures described previously.

3,453 ALSPAC mother and fathers and 535,478 SNPs were genotyped using the Illumina HumanCoreExome chip genotyping platforms by the ALSPAC lab and called using GenomeStudio. The resulting raw genome-wide data were subjected to standard quality control methods using PLINK (v1.07). Individuals were excluded on the basis of gender mismatches (n = 80); minimal or excessive heterozygosity (n = 64); disproportionate levels of individual missingness (>5%, n = 60) and possible contamination (n = 3). Population stratification was assessed by multidimensional scaling analysis and compared with 1000 Genomes phase 3 data and principal component analysis (n = 266); all individuals with non-European ancestry were removed. Cryptic relatedness was measured as SNP relatedness in GCTA (relatedness > 0.1, n = 69 removed). SNPs with a call rate of < 95% or evidence for violations of Hardy-Weinberg equilibrium (P < 1E-7) and those which failed GenomeStudio

quality control measures were removed (n = 21,298). 6,594 duplicate SNPs were also removed.

Data was phased for 3074 samples that passed QC but contained related subjects in SHAPEIT v2.r837. The following were then removed: 155,336 monomorphic SNPs, 1033 markers not in 1000 genomes, 11,842 A/T or G/C SNPs and 10 duplicate sites to give 337,732 SNPs on chromosomes 1-23. Of the 329,363 markers on chromosomes 1-22, 298,742 overlapped the reference genome. These were imputed to the 1000 genomes phase 1 version 3 using the Michigan Imputation Server. 1722 eligible fathers remained after QC, exclusion of duplicate subjects and individuals who had withdrawal of consent.

Supplementary Table 1. Neuroticism SNPs identified by Luciano et al. (2018) used to calculate the polygenic scores and proportion of similarity measures in ALSPAC.

* These SNPs were unavailable in the partner genetic data

| **SNP (rsID)** |
| --- |
| rs4653218 |
| rs169235 |
| rs7578651 |
| rs2678897 |
| rs78323352 |
| rs2042555 |
| rs7567451 |
| rs10497655 |
| rs4673866 |
| rs6773869 |
| rs1542212 |
| rs189298483 |
| rs1282545 |
| rs6791611 |
| rs75976 |
| rs4585149 |
| rs59143394 |
| rs7696796 |
| rs1422192 |
| rs7723944 |
| rs2269426 |
| rs9398586 |
| rs240764 |
| rs11759026 |
| rs2056477* |
| rs57506017 |
| rs60668206 |
| rs802425 |
| rs2690837 |
| rs13239186 |
| rs4731328 |
| rs76335349 |
| rs2407746 |
| rs2921036 |
| rs117374667 |
| rs73190080 |
| rs2953805 |
| rs80279740 |
| rs4841132 |
| rs17662402 |
| rs77156030 |
| rs76333288 |
| rs35169606 |
| rs79487346 |
| rs6982308 |
| rs6601444 |
| rs17711777 |
| rs7005884 |
| rs192083738 |
| rs10097870 |
| rs7814925 |
| rs2380937 |
| rs72694263 |
| rs72700239* |
| rs10757410 |
| rs3793577 |
| rs1521732 |
| rs7857183 |
| rs60150206 |
| rs2149351 |
| rs2683653 |
| rs860626 |
| rs34862781 |
| rs297346 |
| rs2071754 |
| rs7107356 |
| rs10896636 |
| rs496939 |
| rs10789929 |
| rs72995548 |
| rs11214589 |
| rs7111031 |
| rs4936277 |
| rs11605020* |
| rs6606710 |
| rs10507274 |
| rs3741475 |
| rs11068926 |
| rs61361413 |
| rs9572015 |
| rs9541687 |
| rs4772079 |
| rs1275411 |
| rs4140799 |
| rs112850127 |
| rs12896360 |
| rs12441402 |
| rs1563245 |
| rs76064345 |
| rs7175083 |
| rs8039690 |
| rs4362360 |
| rs8063603 |
| rs3785232 |
| rs1870293 |
| rs1050846 |
| rs1109451 |
| rs199534 |
| rs2244497 |
| rs7502590 |
| rs77484855 |
| rs11082011 |
| rs72899043 |
| rs4267411 |
| rs56403421 |
| rs11152363 |
| rs8100891 |
| rs4911448 |
| rs11090045 |
